# Coronary artery calcium score: old faithful delivers again

**DOI:** 10.1007/s12471-019-01361-5

**Published:** 2019-12-21

**Authors:** A. Dedic, J. J. Piek

**Affiliations:** 1 Department of Cardiology, Erasmus MC University Medical Centre, Rotterdam, The Netherlands; 2 Department of Cardiology, Academic Medical Centre, University of Amsterdam, Amsterdam, The Netherlands

In the previous issue of the *Netherlands Heart Journal*, Rijlaarsdam-Hermsen et al. describe the prognostic value of the coronary artery calcium (CAC) score in patients with suspected stable coronary artery disease [1]. In their study, the researchers correlated clinical outcome with the CAC scores of 644 stable chest pain patients who underwent CAC scoring as part of their diagnostic work-up. Their results show that increasing CAC scores are associated with an increased risk of mortality and adverse cardiac events.

A large body of literature has accumulated since the introduction of CAC scoring in the late 1980s [2]. Large, multicentre studies have established the strong prognostic value of CAC scoring in asymptomatic individuals [3, 4]. Absence of CAC, in particular, correlates with a very favourable outcome. In a meta-analysis including more than 70,000 study participants, absence of CAC was correlated with an event rate of less than 0.5% during a follow-up of 4 years [5]. This ‘power of zero’ was also encountered in a symptomatic population, albeit with a slightly higher incidence of cardiovascular events (i.e. 1.8%). Given the excellent prognosis, the additional value of further diagnostic testing of symptomatic patients in the absence of CAC can be debated, especially those with a low pre-test probability and longstanding complaints that correspond to a steady plaque build-up with calcification [6]. Once CAC is present, however, the story becomes more complicated with regard to how to apply the CAC results in clinical management. It is evident that an increase in CAC concurs with a progressively atherosclerotic state and a worse clinical outcome [7]. Symptomatic patients with evidence of coronary artery disease should in any case receive pharmacotherapeutic treatment and risk factor modulation, although this has not been proven specifically for increased CAC scores [8, 9]. The question remains who deserves further investigation and a referral to the catheterisation laboratory. Those that remain symptomatic despite optimal medical therapy (OMT) are logical candidates. However, what about those with subsided chest pain after OMT but elevated CAC scores? Although symptom relief has been achieved, they may have lesions that warrant treatment for prognostic purposes. CAC scoring does not visualise non-calcified plaques or provide information on stenosis severity or specific high-risk plaque features such as positive remodelling and low-attenuation plaque. The risk of missing an important finding such as a significant left main coronary artery stenosis is lurking. With the ever-decreasing radiation gap between CAC scoring and contrast-enhanced coronary computed tomography angiography (CCTA), it is becoming more appealing to proceed with CCTA whilst the patient is on the CT table. The results of the International Study of Comparative Health Effectiveness with Medical and Invasive Approaches (ISCHEMIA) might shed light on this matter [9]. This large multicentre, randomised study compared an invasive approach including invasive angiography to a conservative approach with OMT alone in more than 5,000 symptomatic patients and moderate to severe myocardial ischaemia. All patients underwent CCTA to unmask high-risk patients with a left main coronary artery stenosis and low-risk patients with no obstructive coronary artery disease. Hopefully, the investigators of the ISCHEMIA trial included CAC scoring as part of the CT acquisition protocol. Having the combined results of CAC scores, CCTA and clinical outcome of all patients in this pivotal trial will further define the role of CAC in a symptomatic population.
